# Transcriptomic profiling and discovery of key genes involved in adventitious root formation from green cuttings of highbush blueberry (*Vaccinium corymbosum* L.)

**DOI:** 10.1186/s12870-020-02398-0

**Published:** 2020-04-25

**Authors:** Haishan An, Jiaying Zhang, Fangjie Xu, Shuang Jiang, Xueying Zhang

**Affiliations:** 1 Forestry and Pomology Research Insitute, Shanghai Academy of Agricultural Sciences, Jinqi Road No. 1000, Fengxian District, Shanghai, 201403 China; 2 Shanghai Key Lab of Protected Horticultural Technology, Shanghai Academy of Agricultural Sciences, Jinqi Road No. 1000, Fengxian District, Shanghai, 201403 China

**Keywords:** *Vaccinium corymbosum* L., Adventitious rooting, Differentially expressed genes, Transcriptome analysis

## Abstract

**Background:**

Propagation of cuttings is frequently used in various plant species, including blueberry, which shows special root characteristics that may hinder adventitious root (AR) formation. AR formation is influenced by various factors, and auxin is considered to play a central role; however, little is known of the related regulatory mechanisms. In this study, a comparative transcriptome analysis of green cuttings treated with or without indole-butyric acid (IBA) was performed via RNA_seq to identify candidate genes associated with IBA-induced AR formation.

**Results:**

Rooting phenotypes, especially the rooting rate, were significantly promoted by exogenous auxin in the IBA application. Blueberry AR formation was an auxin-induced process, during which adventitious root primordium initiation (rpi) began at 14 days after cutting (DAC), root primordium (rp) was developed at 21 DAC, mature AR was observed at 28 DAC and finally outgrowth from the stem occurred at 35 DAC. Higher IAA levels and lower ABA and zeatin contents might facilitate AR formation and development. A time series transcriptome analysis identified 14,970 differentially expressed genes (DEGs) during AR formation, of which there were 7467 upregulated and 7503 downregulated genes. Of these, approximately 35 candidate DEGs involved in the auxin-induced pathway and AR formation were further identified, including 10 auxin respective genes (*ARFs* and *SAURs*), 13 transcription factors (*LOB domain-containing protein* (*LBDs*)), 6 auxin transporters (*AUX22*, *LAX3/5* and *PIN-like 6* (*PIL6s*)) and 6 rooting-associated genes (*root meristem growth factor 9* (*RGF9*), *lateral root primordium 1* (*LRP1s*), and *dormancy-associated protein homologue 3* (*DRMH3*)). All these identified DEGs were highly upregulated in certain stages during AR formation, indicating their potential roles in blueberry AR formation.

**Conclusions:**

The transcriptome profiling results indicated candidate genes or major regulatory factors that influence adventitious root formation in blueberry and provided a comprehensive understanding of the rooting mechanism underlying the auxin-induced AR formation from blueberry green cuttings.

## Background

Blueberry (*Vaccinium corymbosum* L.) is a member of the Ericaceae family and a commercially important small fruit crop because of their healthy and flavourful bioactive compounds, and blueberry acreage has been continuously expanded year-by-year worldwide, especially in China [1]. This plant can be propagated by multiple methods, such as seeds, grafting, tissue culture and cuttings, with cuttings primarily used because it can ensure the characteristics of the mother plants and increase plant uniformity [2–5]. Adventitious root (AR) formation is considered a prerequisite for successful propagation of blueberry cuttings. However, due to the special root architecture of blueberry, which mainly consists of fine roots, the cultivation of blueberry usually requires certain environmental conditions, e.g., soil moisture, permeability and pH, which usually lead to a lower adventitious rooting percentage [6, 7]. Until now, the difficulty of blueberry propagation using cuttings has represented a main factor limiting its expansion [8]. Thus, the environmental or genetic mechanisms that control blueberry AR formation must be revealed.

AR formation is a complex developmental process that reflects the plasticity of plants to adjust to stressful conditions and regenerate plant tissues from the same individuals independent of sexual reproduction [9–12]. ARs are usually generated spontaneously or in response to certain stimuli from stems, leaves, or non-pericycle tissues of older roots [13, 14], and it can be divided into several stages based on their physiological and metabolic processes: a) dedifferentiation; b) cell division; and c) adventitious root primordia initiation, development and outgrowth [15]. Plant hormones, including auxin, abscisic acid, cytokinin and ethylene, have been proven to play vital roles in enhancing AR formation, and auxin is considered a central player [16, 17]. Although indole-3-acetic acid (IAA) is a primarily native auxin in plants, synthetic auxin indole-butyric acid (IBA) is more effective in promoting adventitious rooting quality and frequently exogenously applied to promote AR emergence from cuttings of difficult-to-root plant species, including blueberry [6]. For instance, blueberry hardwood or softwood cuttings treated with IBA showed significantly better rooting ability than that of controls [7, 18]. However, limited knowledge is available about the regulatory mechanisms that occur in cuttings after IBA treatment, especially the auxin signalling cascade and auxin-induced gene transcriptional information, during the onset of AR initiation and thus the formation of rooting cues of IBA-treated cuttings. With the rapid development of biological information technology, Illumina sequencing technology (RNA-seq) provides a new gateway to identify the gene expression patterns, regulatory networks and even SNP variants involved in complex biological processes of plants [19–22]. RNA-seq technology is a highly efficient, widely used and conventional molecular biology method for obtaining transcriptomic information and has been successfully applied in blueberry to identify candidate genes involving agronomic traits, such as the putative genes related to antioxidants [19, 23], fruit development and ripening [24, 25], and genes involved in the chilling-mediated flowering pathway [26]. However, no sequence transcriptional information was available for AR formation from cuttings of blueberry.

We proposed a hypothesis that exogenous IBA is involved in the regulation of AR formation by disturbing the balance of endogenous hormones and the genes associated with plant hormone signal transduction, especially in auxin homeostasis, would be mostly affected by IBA application. Therefore, in this study, the root phenotypes, including the rooting percentage, average root number and root length, were analysed to assess the adventitious rooting ability of blueberry green cuttings. The anatomical structure of the stem base was observed to monitor the developmental process of ARs. Dynamic changes in endogenous hormone levels were determined to analyse the effects of hormones on AR formation. Furthermore, a comparative transcriptome analysis of cuttings treated with or without IBA was performed via RNA_seq technology to identify candidate genes involved in the IBA-induced formation of ARs in cuttings and to obtain deeper insights into the mechanisms or regulatory networks that control adventitious rooting events of blueberry cuttings. The results provide a genetic resource for identifying the specific genes and proteins involved in AR formation as well as for improving woody plant propagation of blueberry.

## Results

### Phenotypic analysis

**Fig. 1:**
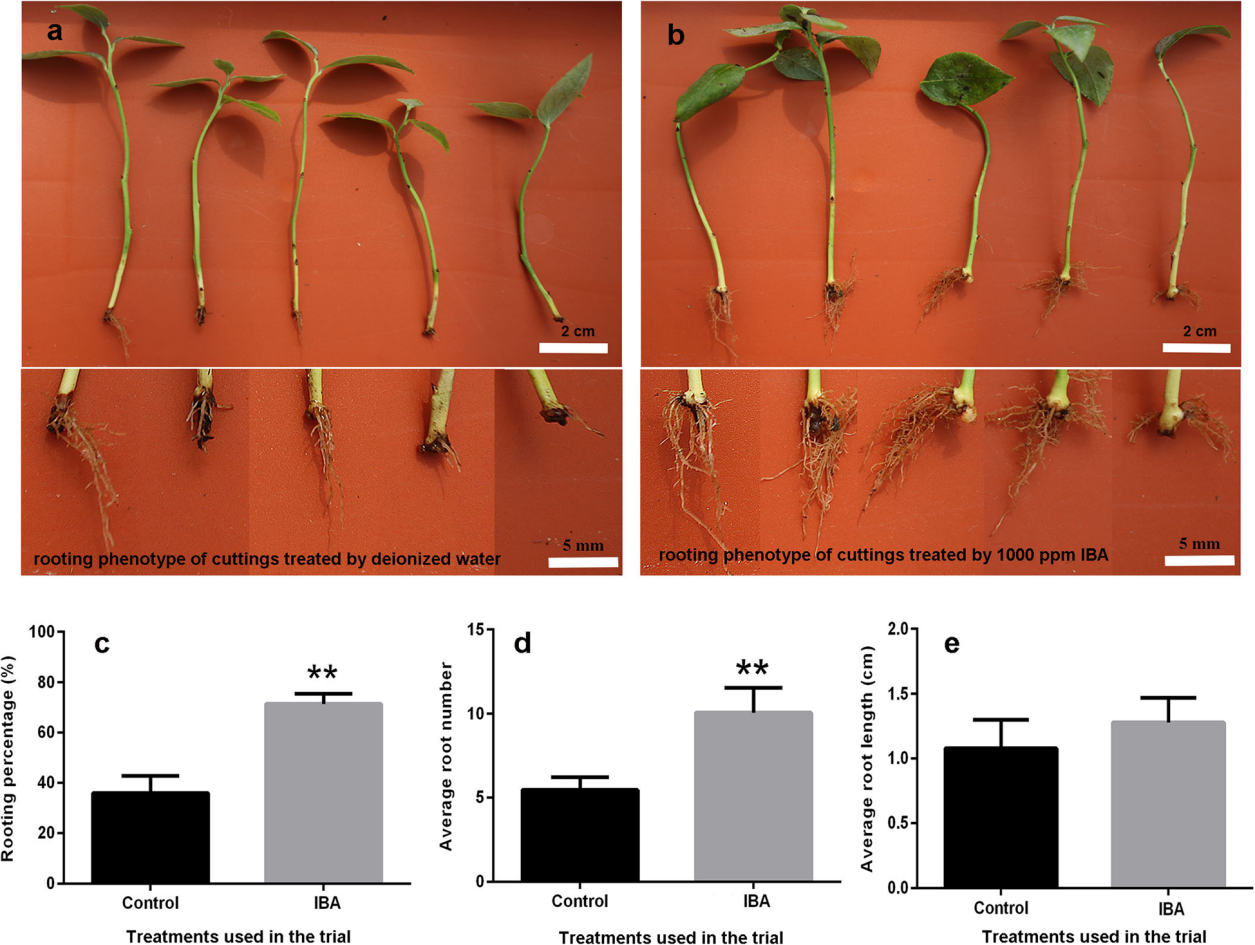
Rooting phenotypes of the stem of green cuttings of blueberry cv. ‘Biloxi’ treated with deionized water (control) and 1000 ppm indole-butyric acid (IBA). Note: **a**, rooting phenotype of green cuttings of ‘Biloxi’ under the control treatment; **b**, rooting phenotype of green cuttings of ‘Biloxi’ under the IBA treatment; **c**, rooting percentage of green cuttings of ‘Biloxi’ under the control and IBA treatment; **d**, average root number per cutting of green cuttings of ‘Biloxi’ under the control and IBA treatment; and **e**, average root length of green cuttings of ‘Biloxi’. Control and IBA indicated the treatment used in this study. Axis X indicates the treatments. All data are the mean value of three replications, the bar indicates standard error; and ** indicates the significance at *P* ≤ 0.01

The rooting phenotype of blueberry green cuttings varied significantly between the control and IBA treatment (Fig. 1a-b). The rooting percentage and average root number per cutting were both significantly promoted by exogenously applied IBA (Fig. 1c-d). There was no significant difference in average root length between these two treatments (Fig. 1e), suggesting that exogenous auxin IBA could enhance adventitious root formation without influencing the average length.

### Microstructure observation of adventitious root formation

**Fig. 2:**
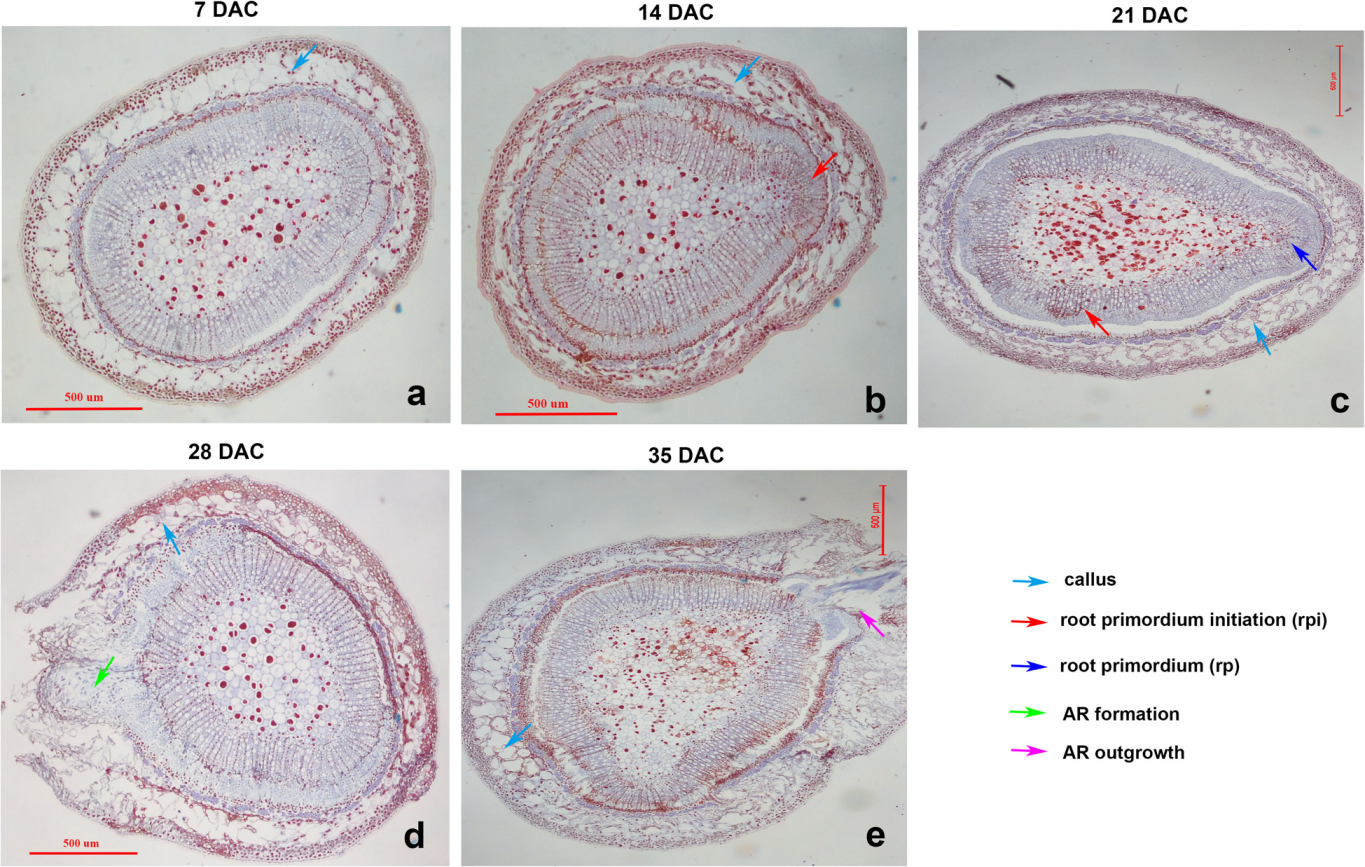
Anatomy of the stem of blueberry green cuttings during AR development. Note: **a**, anatomy of blueberry green cuttings at 7 days after cutting (DAC); **b**, anatomy of blueberry green cutting at 14 DAC; **c**, anatomy of blueberry green cuttings at 21 DAC; **d**, anatomy of blueberry green cuttings at 28 DAC; and **e**, anatomy of blueberry green cuttings at 35 DAC. The bar indicates the scale at 500 um

To clearly and definitely observe the AR developmental process against the stem of blueberry green cuttings, the microstructure was observed. The results showed that callus tissues started to form at 7 days after cutting (DAC) in the IBA treatment (Fig. 2a), adventitious root primordium initiation (rpi) was produced at 14 DAC (Fig. 2b), and adventitious root primordium (rp) developed at 21 DAC (Fig. 2c), rp then developed to adventitious root (AR) (Fig. 2d) and outgrowth from the stem was observed at 35 DAC (Fig. 2e). This process suggests that blueberry AR formation was initiated from non-root pericycle cells as a type of auxin-induction.

### Analysis of plant hormones in blueberry green cuttings during AR formation

**Fig. 3:**
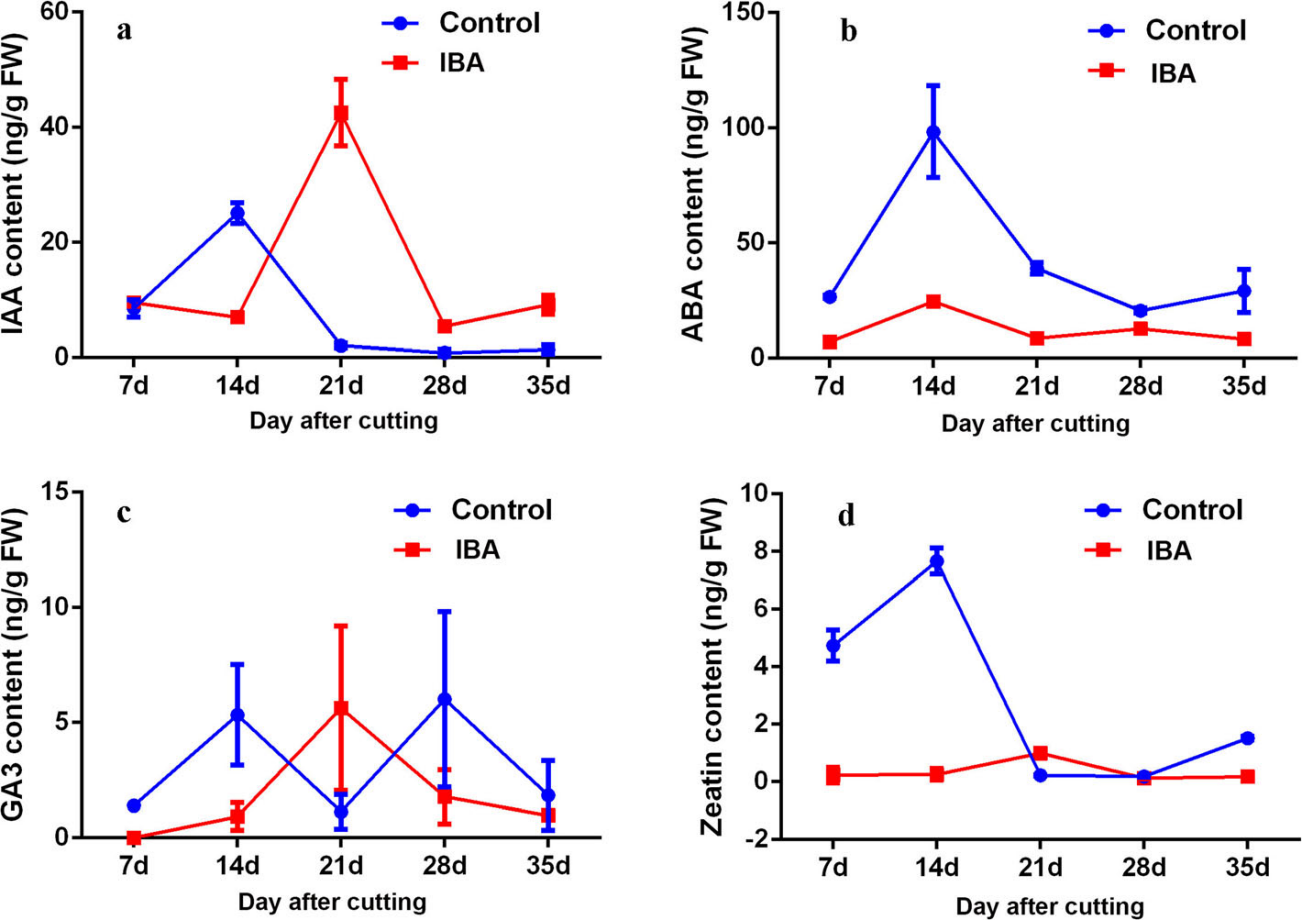
Dynamic changes in endogenous hormones in blueberry green cuttings during AR formation. Note: **a**, changes in indoleacetic acid (IAA) during blueberry AR formation; **b**, changes in abscisic acid (ABA) during blueberry AR formation; **c**, changes in GA3 during blueberry AR formation; and **d**, changes in zeatin during blueberry AR formation. Axis X represents the sampled time, e.g., 7d indicates samples were collected at 7 days after cutting. Control and IBA indicate the treatment used in this study. The bar indicates the standard error (*n* = 3)

Changes in plant hormones during blueberry AR formation were analysed. The level of IAA exhibited a significant increase at 21 DAC under the IBA treatment (Fig. 3a-b), which might indicate that the higher IAA contributes to initiating adventitious root primordium (rpi). No significant difference in GA3 content was observed in the green cuttings between the control and IBA treatment (Fig. 3c). The content of ABA and zeatin under the IBA treatment was kept at a lower level during the whole trial, and a decreasing tendency of ABA and zeatin was recorded in the control treatment and obtained a similar level relative to the IBA treatment after 21 DAC (Fig. 3d), suggesting that a lower level of ABA and zeatin may facilitate AR formation.

### De novo sequencing, assembly, and gene annotation in the RNA-seq analysis of blueberry stem

The basal stem of the cuttings were sampled every 7 days after insertion, and ten samples (CK7, CK14, CK21, CK28, CK35, T7, T14, T21, T28 and T35) were subjected to total RNA extraction and RNA-seq analysis. Total 40.73–46.23 million (M) pairs of 150 bp raw reads were generated from RNA_sequencing for each library (Table 1). After the data filtering, 212 million clean reads remained, out of which the clean reads number for each library ranged from 41.27 to 44.25 M (Table 1). Finally, 672,606 transcripts and 308,719 unigenes were de novo assembled with an average length of 735.87 bp (N50 = 1082) and 617.30 bp (N50 = 844), respectively (Table 2). After Blastx searching with a cut-off *E*-value of 10^− 5^ against five databases, and the total unigenes annotated by NR database, GO database, KEGG database, eggNOG database and Swiss-Prot database were 107,111, 53,859, 6696, 101,729 and 89,910, respectively (Table 3).

**Table 1:** Summary of IIlumina transcriptome sequencing for green cuttings of blueberry

Samples	Raw reads (bp)	Clean reads (bp)	Q20 (%)	Q30 (%)	N percentage
CK7	44,459,682	44,246,320	97.03	92.88	0.00%
CK14	42,676,938	42,497,314	97.16	93.15	0.00%
CK21	43,466,804	43,256,880	97.05	92.90	0.00%
CK28	41,456,090	41,271,800	97.09	93.01	0.00%
CK35	43,865,958	43,657,612	97.06	92.93	0.00%
T7	42,921,102	42,763,116	97.28	93.41	0.00%
T14	46,198,394	45,959,600	96.85	92.53	0.00%
T21	42,930,992	42,735,594	97.10	93.02	0.00%
T28	46,227,958	46,005,308	96.93	92.72	0.00%
T35	40,733,654	40,602,334	97.56	94.00	0.00%

**Table 2:** Length distribution of assemble unigenes

	Contig	Transcript	Unigene
Total length (bp)	258,246,737	494,949,208	190,571,027
Sequence number	854,087	672,606	308,719
Max. length (bp)	23,340	19,233	19,233
Mean length (bp)	302.37	735.87	617.30
N50 (bp)	405	1082	844
N50 sequence No.	135,625	130,694	59,752
GC%	42.24	41.70	42.03

**Table 3:** Summary for the BLASTx results of blueberry transcriptome against five database

Database	Annotated unigene number	Percentage (%)
NR	107,111	34.70
GO	53,859	17.45
KEGG	6696	2.17
eggNOG	101,729	32.95
Swissprot	89,910	29.12
In all database	5184	1.68

Based on NR annotations, 25.33% of the annotated sequences showed very strong homology (*E*-value < 10^− 60^), 21.80% of the annotated sequences showed strong homology (10^− 60^ < *E*-value < 10^− 30^), and an additional 82.87% of the annotated sequences showed homology (10^− 30^ < *E*-value < 10^− 5^) to available plant sequences (Additional file 1: Fig. S1a). The similarity distribution was comparable, with 26.41% of the sequences showing similarities higher than 80 and 73.5% of the hits showing similarities of 0–80% (Additional file 1: Fig. S1b). With respect to species, 72.99% of the unique genes showed high matches with sequences from other species, including *Vitis vinifera*, *Oryza sativa Japonica Group* and *Coffea canephora* (Additional file 1: Fig. S1c).

The GO analysis was performed with Blast2GO software. Out of 308,719 unigenes, 263,143 were classified into the “biological process”, “cellular component” and “molecular function” categories (Additional file 2: Fig. S2). This classification provided some information on the percentage of blueberry unigenes in different signal transduction, catabolic and anabolic processes. For the biological process category, the majority of unigenes was grouped into “metabolic process”, “cellular process” and “single-organism process”, which accounted for approximately 72.83%. In the cellular component category, the unigenes were mainly distributed into “cell”, “cell part”, “membrane”, “membrane part” and “organelle”, accounting for approximately 83.72%. For the molecular function category, a large number of unigenes was distributed into “catalytic activity” and “binding”, which accounted for approximately 86.26% (Additional file 2: Fig. S2).

Based on RSEM quantitative software, the FPKM value of the unigenes was calculated between the control (CK) and IBA treatment (T). The DEGs were identified by pairwise comparisons of ten libraries, i.e., CK7 vs. T7, CK14 vs. T14, CK21 vs. T21, CK28 vs. T28, and CK35 vs. T35 (Additional file 3: Fig. S3a). A total of 14,970 DEGs were determined, out of which 7467 were upregulated and 7503 were downregulated (Additional file 3: Fig. S3b). For each sampled period, 3252 DEGs were detected between CK7 and T7 libraries, and these unigenes were directly affected by IBA treatment and might be associated with callus tissue formation (Fig. 2a, Additional file 3: Fig. S3b). In CK14 vs. T14, 3999 DEGs were identified, which might be related with rpi formation (Fig. 2b, Additional file 3: Fig. S3b). In CK21 vs. T21, 1488 DEGs were identified, which contributed to rp formation (Fig. 2c, Additional file 3: Fig. S3b). In CK28 vs. T28, 2648 DEGs were identified, which contributed to AR formation (Fig. 2d, Additional file 3: Fig. S3b). And in CK35 vs. T35, 3583 DEGs were detected, which might have contributed to AR outgrowth and development (Fig. 2e, Additional file 3: Fig. S3b). However, there were no commonly upregulated or downregulated DEGs at all sampled periods as illustrated in the Venn diagram (Additional file 3: Fig. S3c-d), suggesting that DEGs might play special roles during AR formation, outgrowth and development.

### DEGs enriched in the auxin signalling pathway

**Fig. 4:**
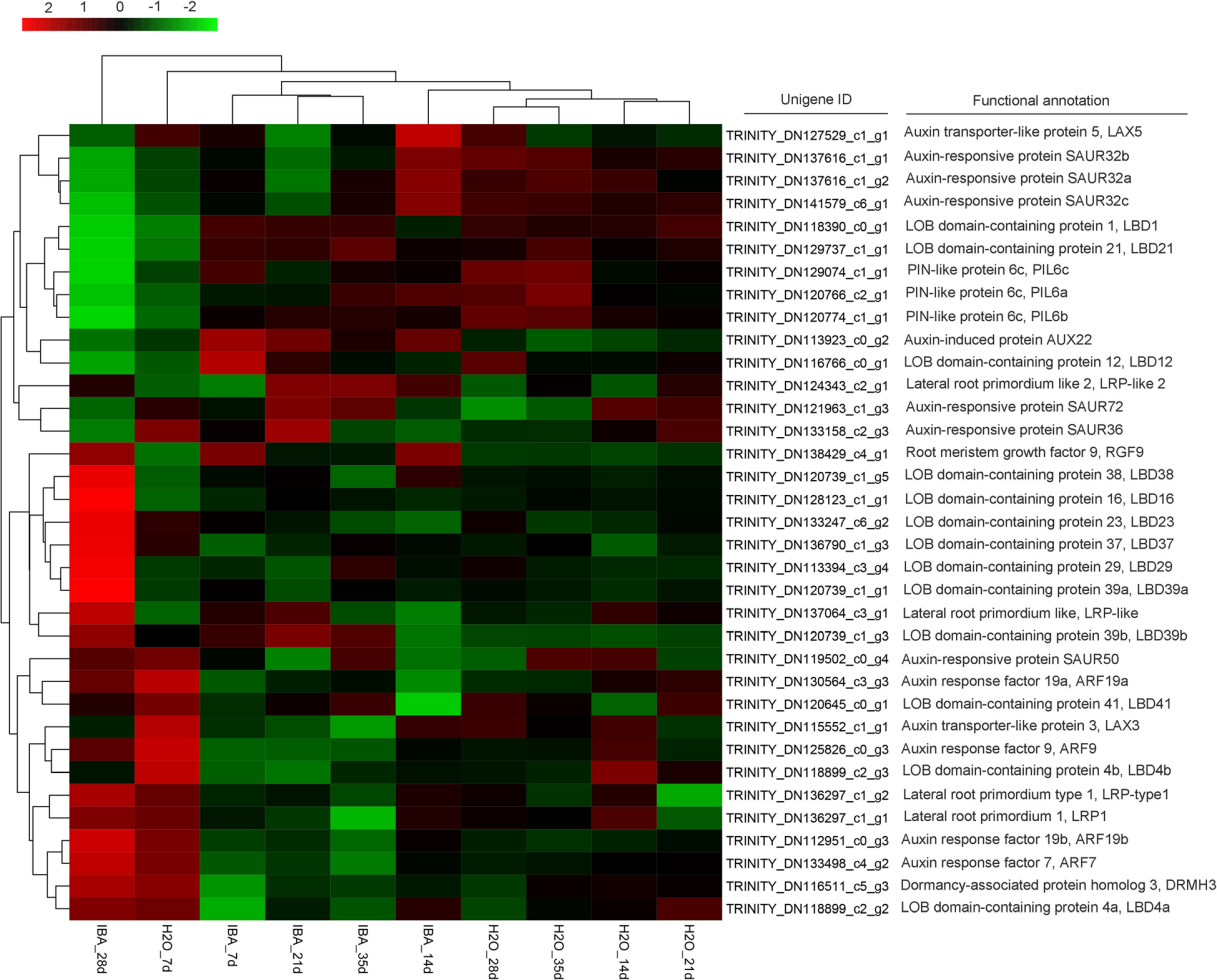
Heatmap of the expression of unigenes related to auxin responsive and root primordium formation in the stem of blueberry green cuttings

**Fig. 5:**
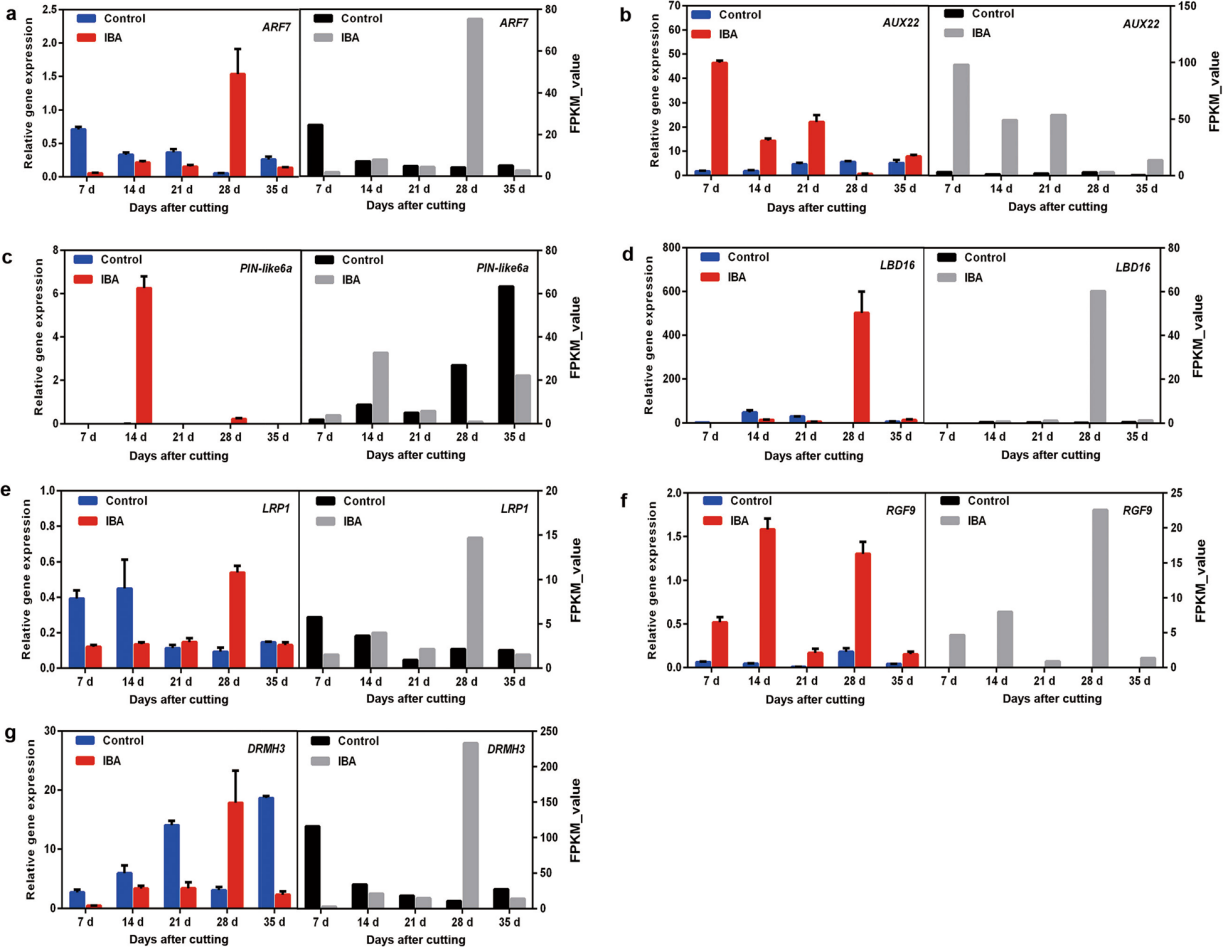
Expression of 7 candidate genes associated with AR formation in the stem of blueberry green cuttings. Note: Control and IBA indicate the treatment used in this study. Axis X indicates the sample time, e.g., 7d means samples were collected at 7 days after cutting; the bar indicates the standard error

Auxin has been proven to play key roles in promoting AR formation; therefore, the DEGs in the auxin-signalling pathway were annotated and further analysed. A total of 29 auxin-related DEGS were mapped (Fig. 4). Of these DEGs, there were ten unigenes belonging to auxin-responsive proteins, including four *ARFs* and six *SAURs*. Six auxin transporter-like genes were also identified, including three influx carriers (*AUX22*, *AUX-LIKE 3* (*LAX3*), and *LAX5*) and three efflux carriers (*PIN-LIKE 6* (*PIL6s*)). All these DEGs were upregulated during AR development to some extent (Fig. 4). To verify the results of the comparative transcription analysis, two auxin responsive factor *ARF* genes (i.e., *ARF7 and ARF9*) and six auxin transporter genes (i.e., *AUX22*, *LAX3*, *LAX5* and *PIL6a-6c*) were selected to identify the DEG expression profiles by qRT-PCR. RNA_seq showed that *ARF7* and *ARF9* exhibited a significant transcript peak at 28 DAC in the IBA treatment, while both these *ARFs* showed a decreasing trend in the control (Fig. 5a, Additional file 4: Fig. S4). RNA_seq data revealed that *AUX22* was kept at a lower expression in the control but showed a significant upregulation at 7 DAC – 21 DAC in the IBA treatment (Fig. 5b). Its homologous genes *LAX3* and *LAX5* showed a decreasing tendency in the control, which were significantly upregulated at 14 DAC and 28 DAC in the IBA treatment (Additional file 5: Fig. S5). These results suggest that these genes might play an important role in auxin transport and contribute to auxin asymmetrical distribution, which was facilitated to induce the adventitious root primordium initiation at the early stage of AR formation. For the three auxin efflux carriers *PIL6a-6c*, in the control treatment, these three genes showed low expression at 7 DAC – 21 DAC and were upregulated until 28 DAC. However, after applying IBA, *PIL6a* was significantly upregulated at 14 DAC (Fig. 5c) and *PIL6b* and *PIL6c* showed a remarkable increase from 7 DAC and peaked at 35 DAC (Additional file 6: Fig. S6), suggesting that auxin enhances the expression of *PIL6s* and thus promotes AR formation. qRT-PCR data indicated a similarity with transcript information.

Transcription factor *LATERAL ORGAN BOUNDARIES DOMAIN/LOB* domain-containing proteins (*LBDs*) were suggested to regulate AR formation as downstream target genes of the *ARF* family. In the present work, thirteen homologue genes of *LBDs* were identified based on NR annotation (Fig. 4), and the RNA-seq data revealed that all these *LBDs* were up- or downregulated at different stages to a certain extent, especially at 28 DAC. Four out of these 13 *LBDs* (i.e., *LBD16, 23, 29* and *37*) were representatively selected to confirm their expression profiles by qRT-PCR, and the results indicated that *LBD16* and *LBD37* showed good reproducibility with the RNA-seq data. Moreover, the qRT-PCR indicated that *LBD23* was upregulated again at 14 DAC in the IBA treatment and *LBD29* showed continuous upregulation from 21 DAC to 35 DAC in the IBA treatment and was also upregulated at 14 DAC and 35 DAC in the control (Fig. 5d, Additional file 7: Fig. S7).

### DEGs involved in root primordium formation

Based on the NR annotation, six rooting-related DEGs were obtained including four homologue genes of *lateral root primordium 1* (*LRP1*), one putative *root meristem growth factor 9* (*RGF9*), and one *dormancy-associated protein homologue 3* (*DRMH3*) (Fig. 4). The RNA_seq data showed that *LRP1* was significantly upregulated at 28 DAC (Fig. 5e), and its homologous genes *LRP1-like*, *LRP-type1, LRP-like2* exhibited continuous upregulation after 14 DAC and peaked at 28 DAC in the IBA treatment (Additional file 8: Fig. S8). Obvious upregulation of *RGF9* was observed at 7 DAC, 14 DAC and 28 DAC (Fig. 5f), *DRMH3* showed significant upregulation at 28 DAC in the IBA treatment (Fig. 5g). The qRT-PCR analysis of *RGF9* indicated good consistency with the RNA_seq data, and the expression of *DRMH3* in the IBA treatment showed good consistency with the RNA_seq data but was continuously expressed at a high level in the control treatment (Fig. 5g).

### Putative gene regulatory networks that control blueberry AR formation

**Fig. 6:**
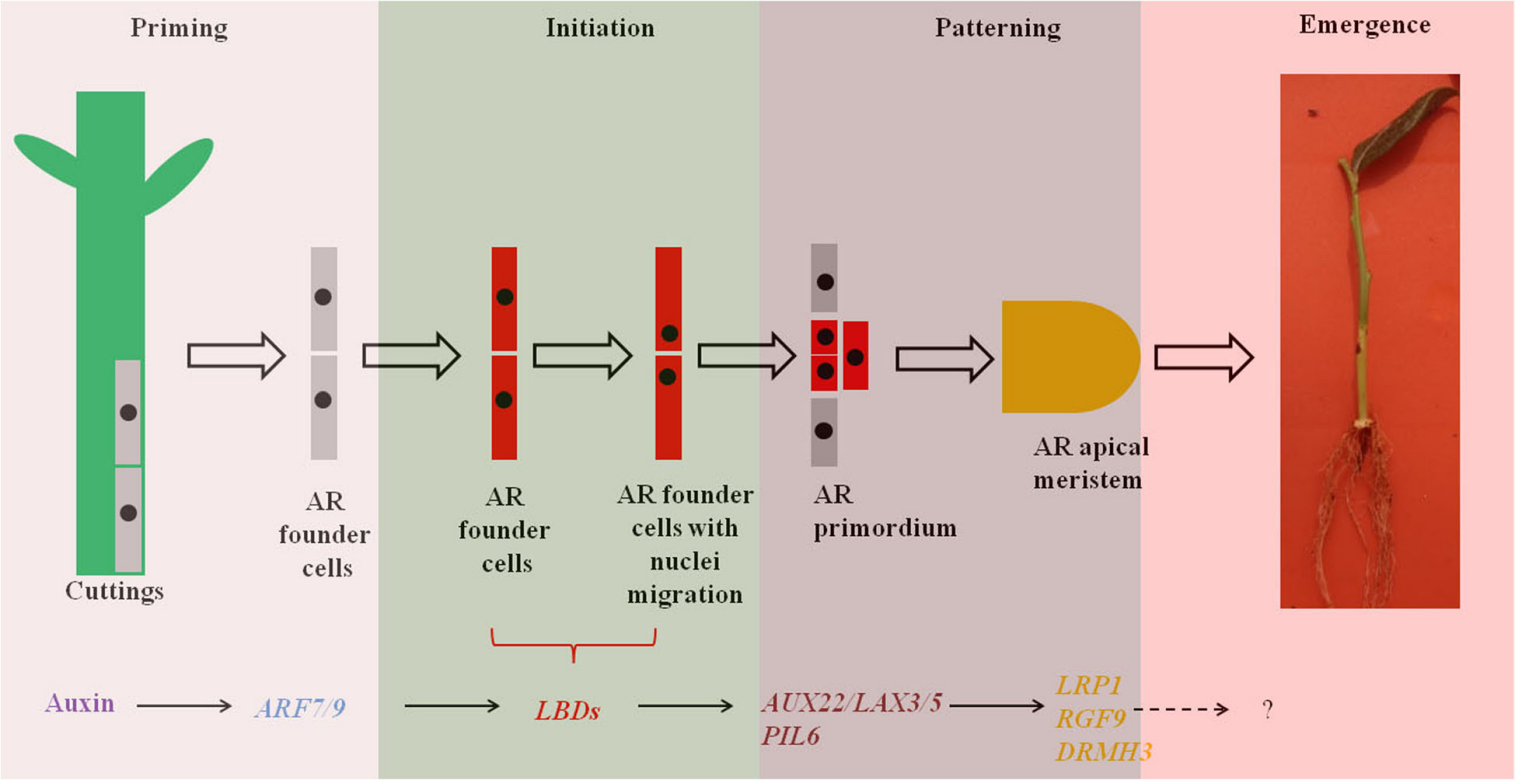
Assumed gene networks that regulate AR formation in blueberry green cuttings. Note: AR, adventitious root; *ARF7/9*, *Auxin responsive factors 7/9*; *LBDs*, Lateral organ boundaries domain; *AUX22*, *Auxin induced protein 22*; *PIL6*, *PIN-LIKE 6*; *LRP1*, *Lateral root primordium 1*; *RGF9*, *root meristem growth factor 9*; and *DRMH3*, *Dormancy-associated protein homologue 3*

According to the known regulatory networks reported previously for root formation in *Arabidopsis* and other plant species, the regulatory pathway that controls blueberry AR formation was derived. It was speculated that IBA would induce the expression of auxin responsive factors *ARF7/9* to perceive auxin signalling, whereas *ARF7/9* directly or indirectly affected the downstream target *LBDs* genes to establish AR founder cells with nuclei migration. Then, auxin polar carriers, including influx carriers *AUX22* or *LAX3/5* and efflux carriers *PIL6s*, would be upregulated to facilitate the establishment of auxin asymmetric distribution, which includes AR primordium formation. Finally, the AR primordium transforms to the AR apical meristem and outgrowth from the cuttings under the effect of *LRP1*, *RGF9*, *DRHM3* and other genes (Fig. 6).

## Discussion

Auxin has been suggested to play a crucial role in promoting the initiation and outgrowth of adventitious roots [27, 28]; however, limited data are available detailing the molecular mechanism about how auxin regulates the adventitious rooting cues of blueberry cuttings. In a previous study, we found that the adventitious rooting ability of blueberry hardwood cuttings depends not only on the cultivar but also on the IBA dosage, with the interaction between the cultivar and IBA contributing to produce better rooting quality [7]. In this work, the adventitious rooting rate of green cuttings treated with IBA was significantly improved. Moreover, AR primordium was initiated after the induction of IBA and the rpi developed into AR and outgrowth from the stem epidermis. These findings were consistent with the suggestion that auxin is an effective inducer of AR formation. The hormonal analysis revealed that the IAA level of the cuttings under the IBA treatment peaked obviously at 21 DAC. This accumulation of IAA likely resulted from the IBA treatment and could accelerate the basal transport of IAA or from a small proportion of absorbed IBA converted into IAA, which finally functions in particular responding cells, initiates self-regulatory auxin canalization and maximization, and thereby starts the process of AR formation [29, 30]. ABA was typically induced during environmental stress and represented an inhibitor of AR formation, and the ABA levels were usually attenuated by IAA so that AR emergence can proceed [31]. The regulatory effect of ABA on AR formation acted indirectly through its dynamic balance with IAA, with a higher ratio of IAA/ABA being conducive to AR formation [32]. In the present work, ABA was maintained at a lower level in the IBA treatment compared to the control, which was similar with the report that IAA-treated stems had the lowest ABA and greatest number of AR [33]. Cytokinins were shown to inhibit AR initiation but positively regulate cell division and stimulate AR elongation [34, 35]. Studies in carnation indicated that cuttings with higher trans-zeatin levels always exhibited a poor adventitious rooting capacity [15, 16]. In this work, the content of zeatin was kept at a very low level in the IBA treatment, which is consistent with previous findings that the cytokinin content in basal parts of cuttings was decreased under IBA treatment [36]. The above results support the hypothesis that IBA affected AR formation by mediating the homeostasis between auxin and other hormones.

Transcriptome analysis is an effective tool to studying gene expression profiles in many biological processes including adventitious rooting [37]. Using this approach, DEGs that respond to AR formation were identified in several other plant species [36, 38]. For blueberries, although adventitious rooting events have been studied for decades, transcript information during AR formation has not been documented. In this work, a comparative transcriptome analysis of blueberry cuttings was employed to identify the unigenes and pathways that were potentially associated with AR formation. In total, more than forty million reads were generated and a total of 672,606 unigenes were assembled. After annotation against the KEGG, GO and other databases, numerous of DEGs involved in the pathway of blueberry AR formation were further identified, indicating that the RNA_seq method was powerful for identifying highly differentially expressed unigenes associated with specific biological process. Furthermore, the presence of these DEGs sheds light on a global view of IBA-induced AR formation in blueberry cuttings, which would facilitate a greater understanding of the molecular mechanisms behind this process and could improve the adventitious rooting efficiency in the agricultural practice of blueberry.

In the model plant *Arabidopsis*, the role of auxin in AR formation was proven to be regulated directly through changes in auxin-related gene expression [39, 40]. Transcriptional regulators *AUXIN RESPONSE FACTOR* (*ARF*s) were demonstrated to be involved in regulating AR formation, for which *ARF*s would activate or repress early responsive genes by banding with auxin response elements in the promoter region of these genes [41]. To date, 29 *ARF*s have been isolated and identified from *Arabidopsis*, out of which *ARF7* and *ARF19* were suggested to participate in AR formation by positively activating the transcription of their downstream genes of *LATERAL ORGAN BOUNDSARIS-DOMAIN* (*LBD*s) [42–44]. Over-expression of *ARF7* and *ARF19* could enhance AR emergence, while the loss-of-function of *arf7*, *arf19* and *arf7/arf19* double mutants led to severe defects in AR formation [42]. The activation of *LBD16* could finally result in the establishment of adventitious root-primordium identity, the *lbd18* mutant exhibited a reduced number of roots and *lbd16lbd18* double mutants showed a dramatic reduction in roots in comparison with *lbd16* or *lbd18*; however, over-expression of *LBD18* rescued the root formation in *lbd18* and *lbd16lbd18* mutant and could stimulate root formation in *arf7arf19* mutants [45–48]. In the present work, a great deal of auxin-induced DEGs, including *ARF7*, *ARF9* and their downstream *LBDs* genes, were identified, and both transcriptome and qRT-PCR data showed obviously upregulation of these DEGS, indicating their potential roles in blueberry AR formation. However, the functional roles of these responsive genes in blueberry AR formation have not been clarified and needs to be verified.

Numerous studies in *Arabidopsis* and other species have indicated that the auxin polar transport (APT) system, which is mediated mainly by influx and efflux carriers, is essential for AR initiation and subsequent development because the APT system is strictly directional to establish auxin asymmetric localization and thus induces ARs initiation and emergence [49–51]. *PIN-FORMED* (*PIN*) protein and its analogous *PIN-LIKES* (*PILS*) were believed to be efflux carriers, *AUX1* and *AUX1-LIKE* (*LAX*) families acted as influx carriers and have been proven to exert great influence on AR formation [52–55]. Upregulation of these efflux carrier genes might have contributed to the observed PAT-dependent auxin peak and thus to the induction or initiation of AR formation [14, 30]. It was previously suggested that higher expression of *PIN1* would enhance AR formation from non-root tissues, with the *pin1* mutant failing to establish an auxin gradient and showing developmental disorders in root formation. Similarly, AR development in rice was significantly inhibited in *OsPIN1* RNA interference (RNAi) transgenic plants, which exhibited a defect in AR emergence, and exogenously applied NAA could rescue the rooting phenotypes in *RNAi-OsPIN1* plants [56]. The *pils2pils5* double loss of function mutant had higher auxin levels and lateral root numbers than the *PILS5* gain-of-function phenotype [57, 58]. An analysis of the localized expression patterns of auxin-induced genes would help reveal the underlying regulatory pathways that control adventitious rooting events in blueberry. In this work, several auxin influx and efflux carrier genes were identified and their expression was found to change vastly with AR development, with the auxin-reduced protein *AUX22* upregulated at a very early stage i.e., 7 DAC - 21 DAC. Moreover, the auxin influx carriers *LAX3/5* and auxin efflux carrier *PIN-like6* were also upregulated, suggesting that these auxin-related genes might participate in the blueberry AR induction phase, in which the founder cells began to be dedifferentiated and then the dome-shaped root primordial was formed [59]. Although the functions of these DEGs have been identified in *Arabidopsis* or other plant species, their functional roles in regulating blueberry AR formation remain unclear and need to be further studied.

*Lateral root primordial 1* (*LRP1*)*,* which is one of ten members of the *SHI* gene family, acts as a transcriptional activator downstream of *AUX/IAA*, and it is expressed mainly in the early stages of lateral root primordium formation [60–63]. *LRP1-like* transcripts were initially detected in the cotyledon and then rapidly became restricted to the upper zone, where cell division and root formation took place [64]. In this work, four *LRP-like* genes were identified by transcriptome data and they were certainly upregulated after IBA treatment, especially at 21 DAC and 28 DAC, during which the rp/rpi and AR begin to be formed, indicating the potential role of *LRP-like* genes in blueberry AR formation. In addition, some other genes, such as root meristem growth factor 9 (*RGF9*) and dormancy-associated protein homologue 3 (*DRMH3*), were identified in this present study. *RGF*, an important peptide hormone, was suggested to regulate root meristem development through its effects on the *PLT1/2* stem cell transcription factor or by regulating of the stability of the receptor *RGFR1* [65, 66]. In the present work, both the transcriptome and qRT-PCR data indicated that *RGF9* was upregulated after IBA treatment. *DRM*, one of auxin-repressed super-family genes, was suggested to be highly expressed in both dormant and non-growing tissues. *BrDRM1* was indicated to be negatively associated with root growth, which was reduced by more than 50% in *BrDRM1*-overexpressing *Arabidopsis* plant, and the reduction was correlated to an increase in *BrDRM1* expression levels [67]. However, in this study, *DRMH3* was highly regulated with AR formation, especially at 28 DAC after IBA treatment. This inconstancy might be caused by the varied function of *DRM* based on the plant species or from the functional differences among each member of the *DRM* family. Therefore, the molecular function of *DRMH3* in blueberry AR formation should be further analysed. Although the molecular functions of the DEGs identified in this study remain unknown, our work offers a foundation for the future characterization of gene functions to ascertain the metabolism of IBA-induced AR formation in blueberry. However, AR formation is a complex developmental process controlled by multiple genes or transcription factors in addition to these DEGs in the auxin signalling pathway; thus, genes in other biological pathways might also play potential roles in regulating AR formation. Therefore, in the future, we will attempt to perform a comprehensive analysis of transcriptome data to broaden our understanding of the regarding regulatory mechanisms involved in AR formation of blueberry, such as the DEGs in the auxin biosynthesis and distribution, secondary metabolism, transport and degradation pathways as well as the DEGs associated with cytokinin, brassinosteroids (BRs), ABA, ethylene, GA and their interactions with the candidate DEGs in the auxin-signalling pathway.

## Conclusions

The adventitious rooting rate was significantly improved by IBA application. AR formation in blueberry was an auxin-induced process, with the adventitious root primordium (rp) initiated at 14 DAC, the rp formed at 21 DAC, the AR formed at 28 DAC and outgrowth from the stem observed at 35 DAC. Higher IAA content and lower ABA or zeatin levels would facilitate blueberry AR formation. To our knowledge, this work was the first to provide a comprehensive transcriptome profiling database for a dynamic view of transcript information in the IBA-induced AR formation in blueberry. A total of 672,606 transcripts and 308,719 unigenes were assembled. Approximately 14,970 DEGs were successfully identified, out of which 7467 were upregulated and 7503 were downregulated. A total of 35 DEGs involved in auxin-signalling and rooting-related pathways were considered as candidate genes based on their expression profiles. Although further research on the functional characteristics of these DEGs is required, our findings offer new insights into the molecular mechanisms underlying blueberry AR formation and provide a relatively complete molecular platform for future studies on the progression of blueberry AR formation. Moreover, the findings in the present work allow for the identification of candidate genes involved in blueberry AR formation and thus represent an important molecular resource for the further fast propagation and breeding of blueberry. Future works should aim to characterize the functional role of these identified individual DEGs and thus their regulatory networks or cross-talks.

## Methods

### Plant material

The southern highbush blueberry cultivar ‘Biloxi’ was used as the material in this study and obtained from the Hongyue Flower Co. LTD, and the plants were then cultivated at the Zhuang-hang Comprehensive Experimental Station of the Shanghai Academy of Agricultural Sciences, Shanghai, China. In spring, approximately 150 green cuttings with a length of 10–15 cm were randomly collected and most of the leaves were removed, retaining the top 1–2 leaves. The cuttings were immersed in deionized water (control) and 1000 ppm indole-butyric acid (IBA, 1 g dissolved in 600 mL 75% alcohol and diluted with water to 1 L) for 1 min and then inserted into growth medium that consisted of peat moss, vermiculite and garden soil in a ratio of 3:1:1 (v/v). Water management was previously described in detail in An et al. (2019) [7]. Approximately 40 days after the trial installation, the rooting phenotypes, including the rooting percentage, average root number and average root length, were surveyed. All experiments were performed in triplicate.

### Analysis of plant hormones in blueberry cuttings

The stem from the base of the cuttings was randomly sampled at a 7-day interval after the trial installation (i.e., stems were sampled at 7 d, 14 d, 21 d, 28 d, and 35 d). Approximately 0.5 g of fresh tissues from each sampled time were ground in liquid nitrogen and digested in 5 mL ethyl acetate for 12 h at 4 °C and then centrifuged at 10000 rpm at 4 °C for 10 min. The residue composition was further digested in 3 mL ethyl acetate and centrifuged at 10000 rpm at 4 °C for 10 min. The supernatant was dried by nitrogen flow at 25 °C, dissolved using 300 μL chromatographic methanol and then ultrasonically extracted for 10 min. Finally, the solution was filtered with a 0.22 μm membrane filter, and 5 μL was injected for analysis. The level of endogenous plant hormones (including IAA, ABA, GA3 and zeatin) was analysed by liquid chromatography-mass spectrometry (LC-MS) according to Niu et al. (2014) [68] with minor modifications. Briefly, the mobile phase consisted of acetonitrile (solvent A) and 0.02% (v/v) glacial acetic acid in water (solvent B). The samples were purified in the C-18 column of the liquid chromatography system (ACQUITY I-CLASS, Waters) and finally determined with mass spectrometry (AB SCIEX analyst, QTRAP™ 5500). Standards of IAA (cas:87–51-4, Sigma), ABA (cas:21293–29-8, Sigma), GA3 (cas:77–06-5, Sigma) and zeatin (cas:13114–27-7, Sigma) were used to optimize the mass spectrometric parameters and fragment spectra. The calibration curves of the IAA, ABA, GA3 and zeatin standards were obtained using six different concentrations (0, 2.5, 5.0 10, 12.5, 25, 50 and 100 ng/mL). The linear regression of the calibration curves is detailed in Additional file 9: Table S1.

### Microstructure observations

After insertion, a 1-cm section from the bottom of the cuttings was sampled at a 7-day interval to observe the formation of adventitious root. All samples were collected from three biological replicates, with *n* = 10 for each replicate. The samples were fixed in the FAA solution (formaldehyde/acetic acid/70% ethanol = 5:5:90, v/v/v). Before microstructure observation, the samples were softened for approximately 10 days by 4% ethylenediamine and then dehydrated using graded ethanol (75, 85, 95, and 100%). Vitrification was performing using dimethylbenzene, and then the samples were embedded with paraffin. Then, 10-μm-thick transverse sections were cut with a rotatory microtome (LEICA, RM2265), and photos were captured and observed under a light microscope (NIKON ECLIPSE E200).

### Total RNA extraction, sequencing and de novo transcriptome assembly

The stem of the base of cuttings (1–2 cm) was sampled at a 7-day interval, and then the samples were immediately frozen in liquid nitrogen and stored in − 80 °C before being analysed. Total RNA was extracted by the TRIzol Reagent (Invitrogen Life Technologies, USA). RNA integrity was then confirmed using the Agilent 2100 Bioanalyzer (Agilent, CA, USA). The RNA-seq libraries were generated with the TruSeq RNA Sample Preparation Kit (Illumina, San Diego, CA, USA), and were applied to a HiSeq platform (Illumina) for RNA_seq analysis by Shanghai Personal Biotechnology Co., Ltd. Transcriptome de novo assembly was carried out using Trinity.

### Sequence annotation and identification of significantly different expressed genes (DEGs)

All unigenes were annotated by BLASTx searchs against five databases, i.e. NR database, GO database, KEGG database, eggNOG database and Swiss-Prot database with *E*-value ≤10^− 5^, and the sequence direction was determined based on the best-aligning results. GO enrichment and the KEGG pathway annotation were obtained using BLAST2GO software and KEGG Automatic Annotation Server, respectively. The significance at *P*-value ≤0.05 of the DEGs were determined by DESeq software with a threshold of |log2FoldChange| > 1 [69–72].

### Gene expression analysis by qRT-PCR

For qRT-PCR, about 1 μg of total RNA were reversed to obtain cDNA using the PrimerScript™ RT reagent Kit with gDNA Eraser (RR047, Takara, Japan). The qRT-PCR were carried out on a LightCycler 480 Real-Time PCR System (Roche, Basal, Switzerland) in a total volume of 10 μl containing 5.0 μl SYBR Premix ExTaq™ (RR820A), 0.5 μl of each primer (10 uM), 2 μl of cDNA and 2 μl ddH_2_O. The PCR conditions were described detail in Jiang et al. (2016) [73]. *GAPDH* was used as internal control. Each reaction was performed with three biological replicates, and each sample was analysed in triplicate (technical replicates). The special primer sequences for the qRT-PCR analysis are shown detail in Additional file 10: Table S2.

### Statistical analysis

The data analysis was performed via a one-way analysis of variance (ANOVA) with SPSS 18.0 software (SPSS Inc., Chicago, USA). Graphics were created using GraphPad Prism 6.0 (GraphPad Software, Inc.).

## Supplementary information

**Additional file 1: Figure S1.** Characteristics of the homology search of assembled blueberry unigenes against the NR database. Note: a, E-value distribution of the top BLAST hits for each unique sequence with a cut-off *E*-value of 1.0 E^− 5^; b, similarity distribution of the top BLAST hits for each unigene; and c, species distribution of all homologous unigenes with an *E*-value of at least 1.0 E^− 5^.

**Additional file 2: Figure S2.** Functional annotation of blueberry based on Gene Ontology. A total of 263,143 unigenes were categorized into three main categories: biological process (96615), cellular component (103924) and molecular function (62604).

**Additional file 3: Figure S3.** Statistics of differently expressed genes (DEGs). Note: a, significantly up- or downregulated genes using the threshold of *P*-value ≤0.001 and log_2_Ratio ≥ 1 in CK28 vs. T28; b, graphical representation of overall differently expressed genes in the IBA treatment; c, number of upregulated unigenes in the IBA treatment illustrated using a Venn diagram; and d, number of downregulated unigenes in the IBA treatment illustrated using a Venn diagram.

**Additional file 4: Figure S4** Expression of auxin responsive factor *ARF9* during AR formation in the stem of blueberry green cuttings. Note: Control and IBA indicate the treatment used in this study. Axis X indicates the sampled time, e.g., 7d means the samples were collected 7 days after cutting. The bar indicates the standard error (*n* = 3).

**Additional file 5: Figure S5.** Expression of auxin influx carriers *LAX3* and *LAX5* during AR formation in the stem of blueberry green cuttings. Note: Control and IBA indicate the treatment used in this study. Axis X indicates the sampled time, e.g., 7d means the samples were collected 7 days after cutting. The bar indicates the standard error (n = 3).

**Additional file 6: Figure S6.** Expression of auxin efflux carriers *PIN-like 6b* and *PIN-like 6c* during AR formation in the stem of blueberry green cuttings. Note: Control and IBA indicate the treatment used in this study. Axis X indicates the sampled time, e.g., 7d means the samples were collected 7 days after cutting. The bar indicates the standard error (n = 3).

**Additional file 7: Figure S7.** Expression of four *LBD*s during AR formation in blueberry green cuttings. Note: Control and IBA indicate the treatment used in this study. Axis X indicates the sampled time, e.g., 7d means the samples were collected 7 days after cutting. The bar indicates the standard error (n = 3).

**Additional file 8: Figure S8.** Expression of four lateral root primordium protein-related *LRP* genes during AR formation in the stem of blueberry green cuttings. Note: Control and IBA indicate the treatment used in this study. Axis X indicates the sampled time, e.g., 7d means the samples were collected 7 days after cutting. The bar indicates the standard error (n = 3).

**Additional file 9: Table S1** Regression equation of the calibration curves for IAA, ABA, GA3 and zeatin.

**Additional file 10: Table S2** Primers for the qRT-PCR analysis used in this study.

## Data Availability

All of the raw data have been deposited in two databases, i.e. NCBI Sequence Read Archive (SRA) with accession number SRR11517305 (CK7), SRR11517304 (CK14), SRR11517303 (CK21), SRR11517302 (CK28), SRR11517301 (CK35), SRR11517300 (T7), SRR11517299 (T14), SRR11517298 (T21), SRR11517297 (T28), SRR11517296 (T35); and the Genome Sequence Archive (GSA) in National Genomics Data Centre under project number CRR078491 (CK7), CRR078492 (CK14), CRR078493 (CK21), CRR078494 (CK28), CRR078495 (CK35), CRR078496 (T7), CRR078497 (T14), CRR078498 (T21), CRR078499 (T28) and CRR078500 (T35). All data in both databases had been released.

## Abbreviations

ABA: Abscisic acid
ANOVA: Analysis of variance
APT: Auxin polar transport
AR: Adventitious root
ARF: Auxin response factor
AUX/IAA: AUXIN/INDOLE-3-ACETIC ACIDS
DAC: Days after cutting
DEGs: Differentially expressed genes
DRMH3: Dormancy-associated protein homologue 3
eggNOG: evolutionary genealogy of genes: Non-supervised Orthologous Groups
FPKM: Fragments per kb per million fragments
GA3: Gibberellin 3
GO: Gene Ontology
GSA: Genome Sequence Archive
IAA: Indole-3-acetic acid
IBA: Indole-butyric acid
KEGG: Kyoto Encyclopaedia of Gene and Genome
LAX: AUX1-LIKE
LBD: LOB domain-containing protein
LC-MS: Liquid chromatography-mass spectrometry
LRP1: Lateral root primordial 1
NR: Non-redundant protein
PILs: PIN-LIKEs
PIN: PIN-FORMED
RGF9: Root meristem growth factor 9
RNAi: RNA interference
rp: root primordium
rpi: root primordium initiation
