# Supplementary figures and images for: Transcriptomic profiling and discovery of key genes involved in adventitious root formation from green cuttings of highbush blueberry (Vaccinium corymbosum L.)
Source: BMC Plant Biol. 2020 Apr 25;20:182. doi: 10.1186/s12870-020-02398-0 (PMC7183619; doi:10.1186/s12870-020-02398-0)

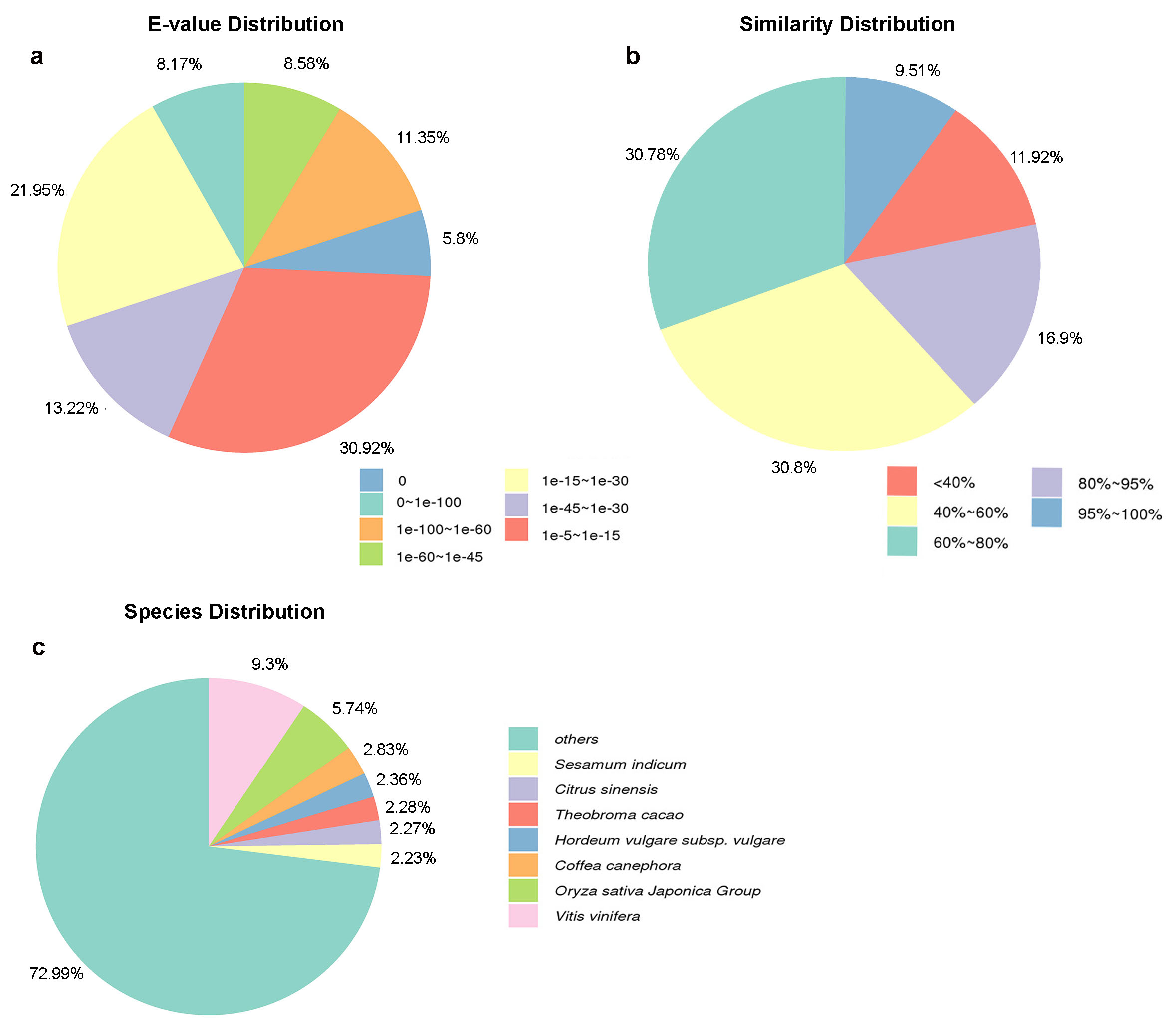

Supplement: Supplementary file 1 — Additional file 1: Figure S1. Characteristics of the homology search of assembled blueberry unigenes against the NR database. Note: a, E-value distribution of the top BLAST hits for each unique sequence with a cut-off E-value of 1.0 E− 5; b, similarity distribution of the top BLAST hits for each unigene; and c, species distribution of all homologous unigenes with an E-value of at least 1.0 E− 5. [file 12870_2020_2398_MOESM1_ESM.jpg]

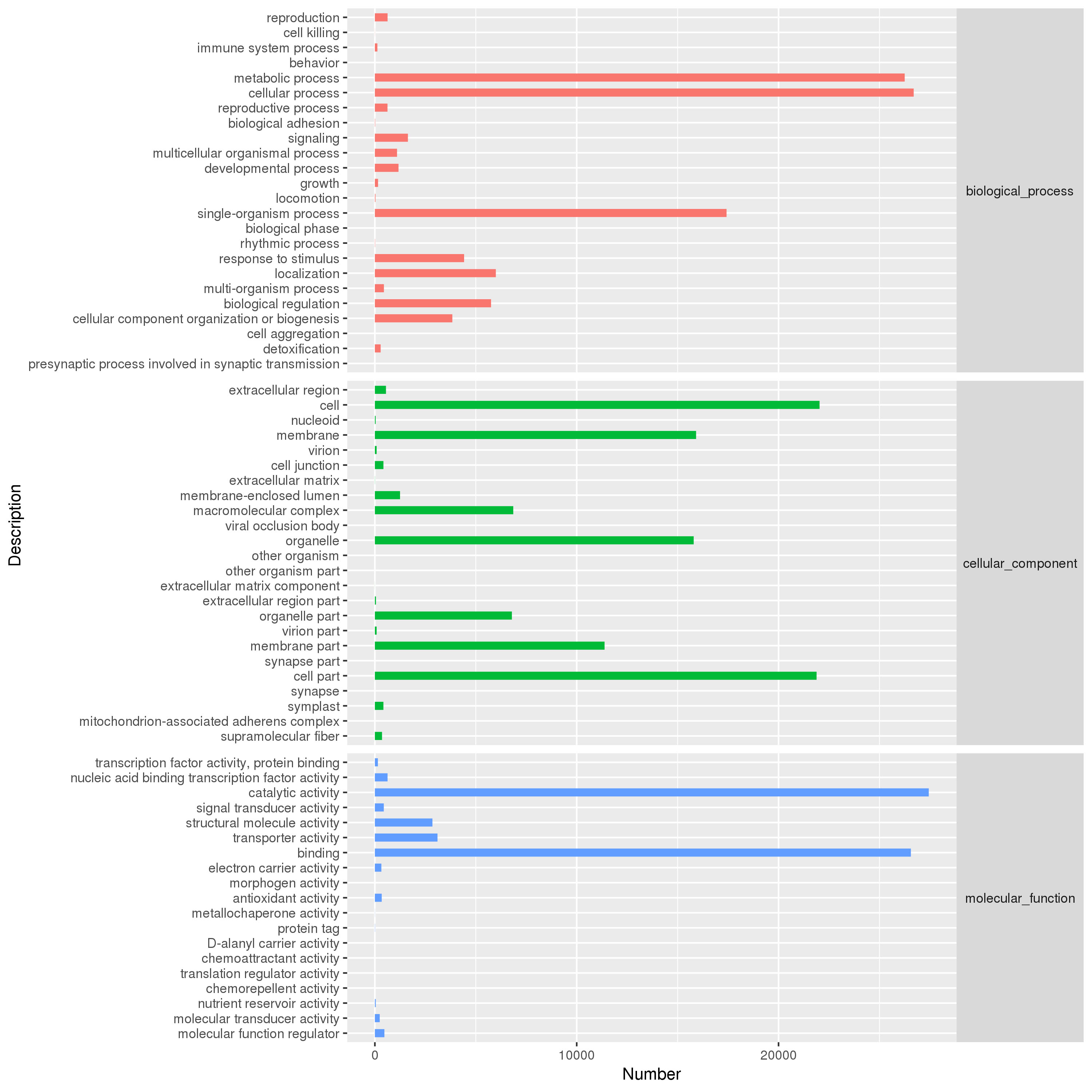

Supplement: Supplementary file 2 — Additional file 2: Figure S2. Functional annotation of blueberry based on Gene Ontology. A total of 263,143 unigenes were categorized into three main categories: biological process (96615), cellular component (103924) and molecular function (62604). [file 12870_2020_2398_MOESM2_ESM.jpg]

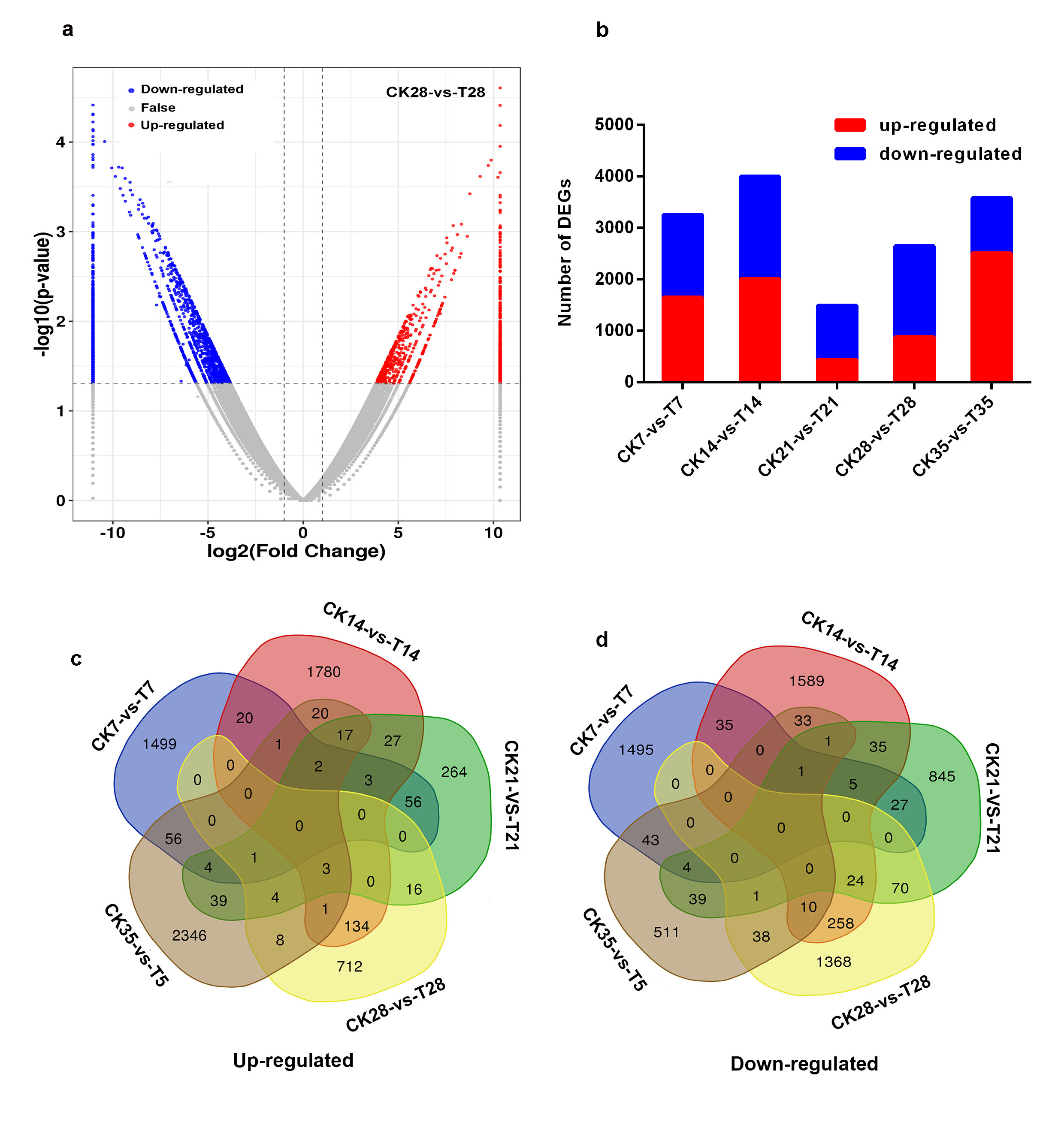

Supplement: Supplementary file 3 — Additional file 3: Figure S3. Statistics of differently expressed genes (DEGs). Note: a, significantly up- or downregulated genes using the threshold of P-value ≤0.001 and log2Ratio ≥ 1 in CK28 vs. T28; b, graphical representation of overall differently expressed genes in the IBA treatment; c, number of upregulated unigenes in the IBA treatment illustrated using a Venn diagram; and d, number of downregulated unigenes in the IBA treatment illustrated using a Venn diagram. [file 12870_2020_2398_MOESM3_ESM.jpg]

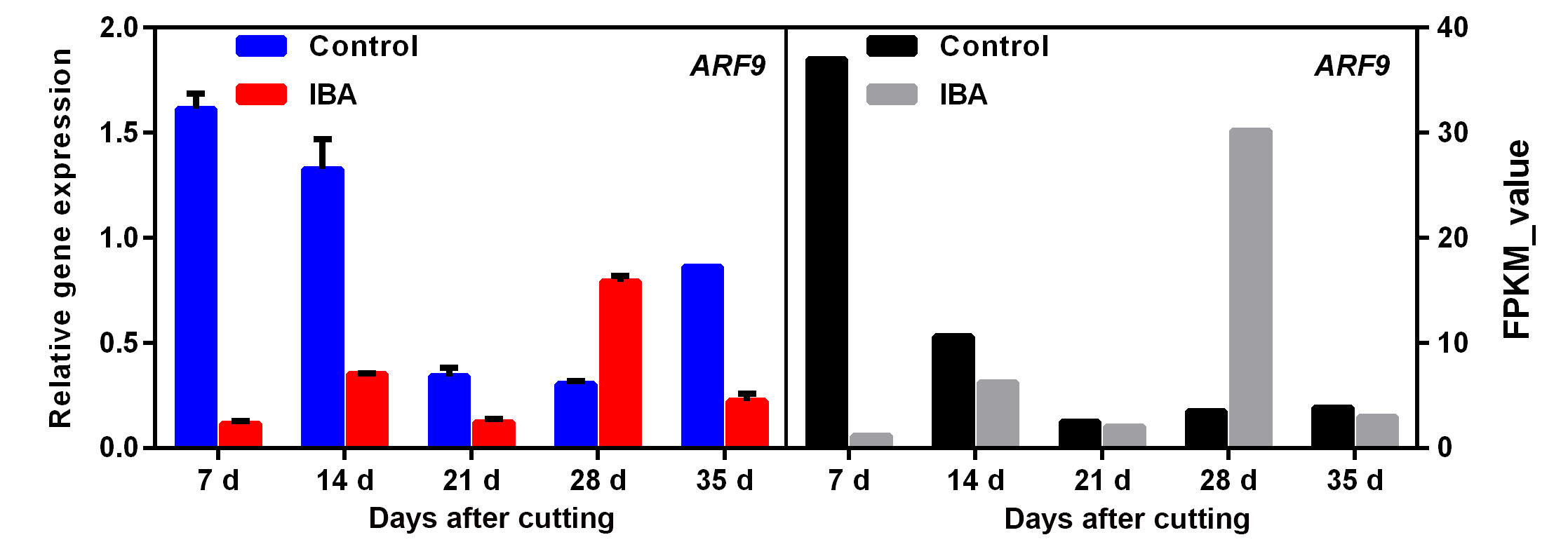

Supplement: Supplementary file 4 — Additional file 4: Figure S4 Expression of auxin responsive factor ARF9 during AR formation in the stem of blueberry green cuttings. Note: Control and IBA indicate the treatment used in this study. Axis X indicates the sampled time, e.g., 7d means the samples were collected 7 days after cutting. The bar indicates the standard error (n = 3). [file 12870_2020_2398_MOESM4_ESM.jpg]

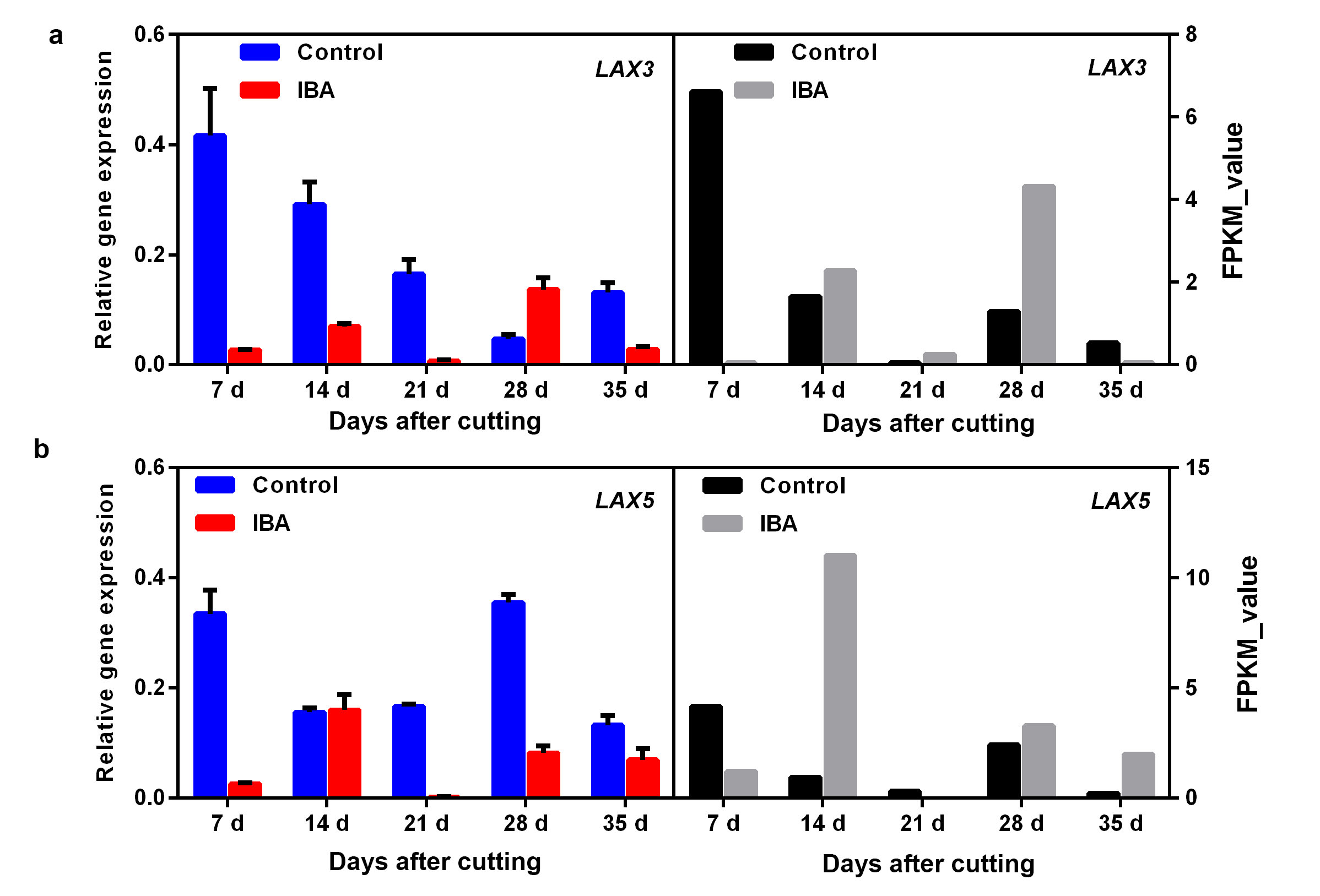

Supplement: Supplementary file 5 — Additional file 5: Figure S5. Expression of auxin influx carriers LAX3 and LAX5 during AR formation in the stem of blueberry green cuttings. Note: Control and IBA indicate the treatment used in this study. Axis X indicates the sampled time, e.g., 7d means the samples were collected 7 days after cutting. The bar indicates the standard error (n = 3). [file 12870_2020_2398_MOESM5_ESM.jpg]

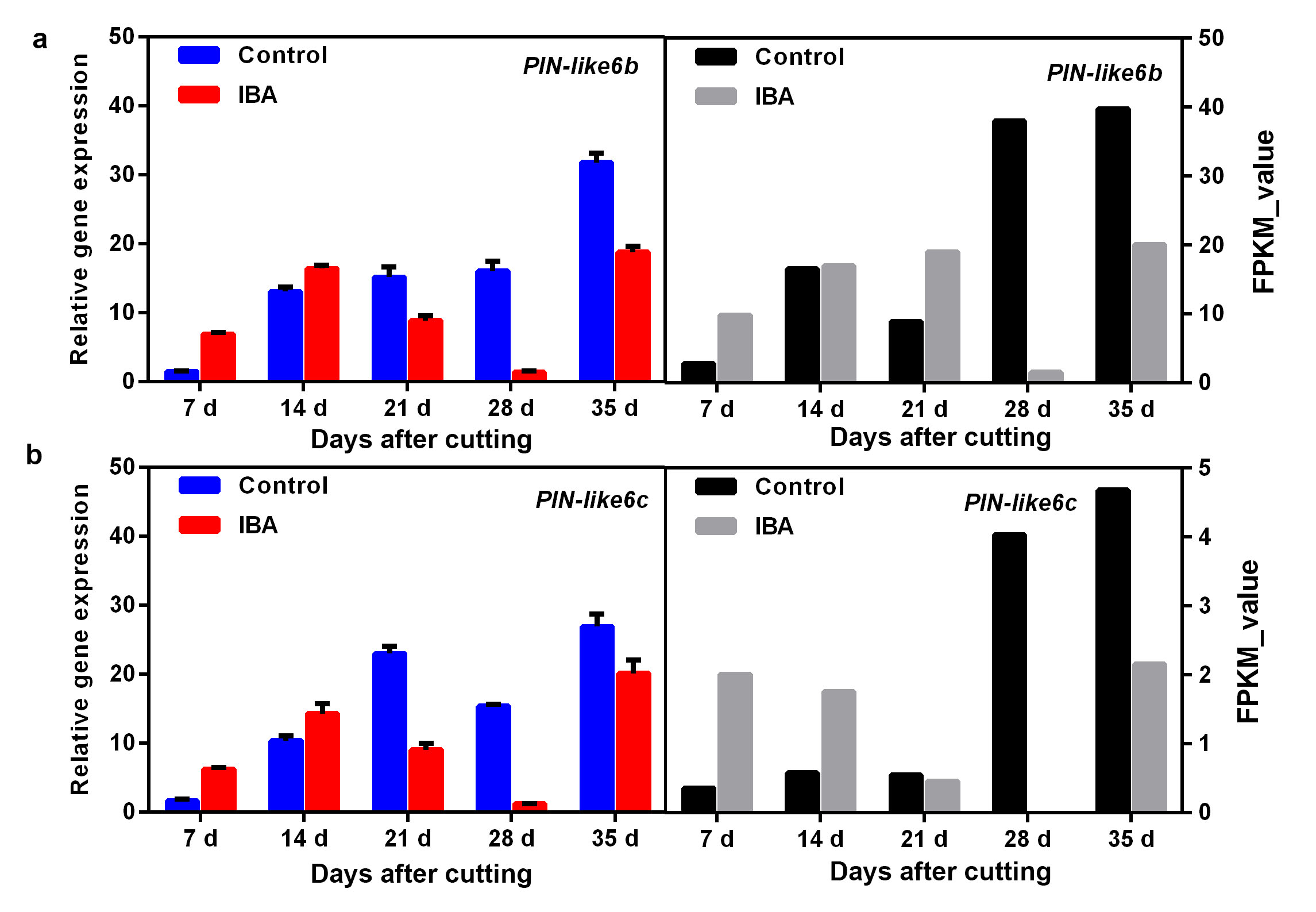

Supplement: Supplementary file 6 — Additional file 6: Figure S6. Expression of auxin efflux carriers PIN-like 6b and PIN-like 6c during AR formation in the stem of blueberry green cuttings. Note: Control and IBA indicate the treatment used in this study. Axis X indicates the sampled time, e.g., 7d means the samples were collected 7 days after cutting. The bar indicates the standard error (n = 3). [file 12870_2020_2398_MOESM6_ESM.jpg]

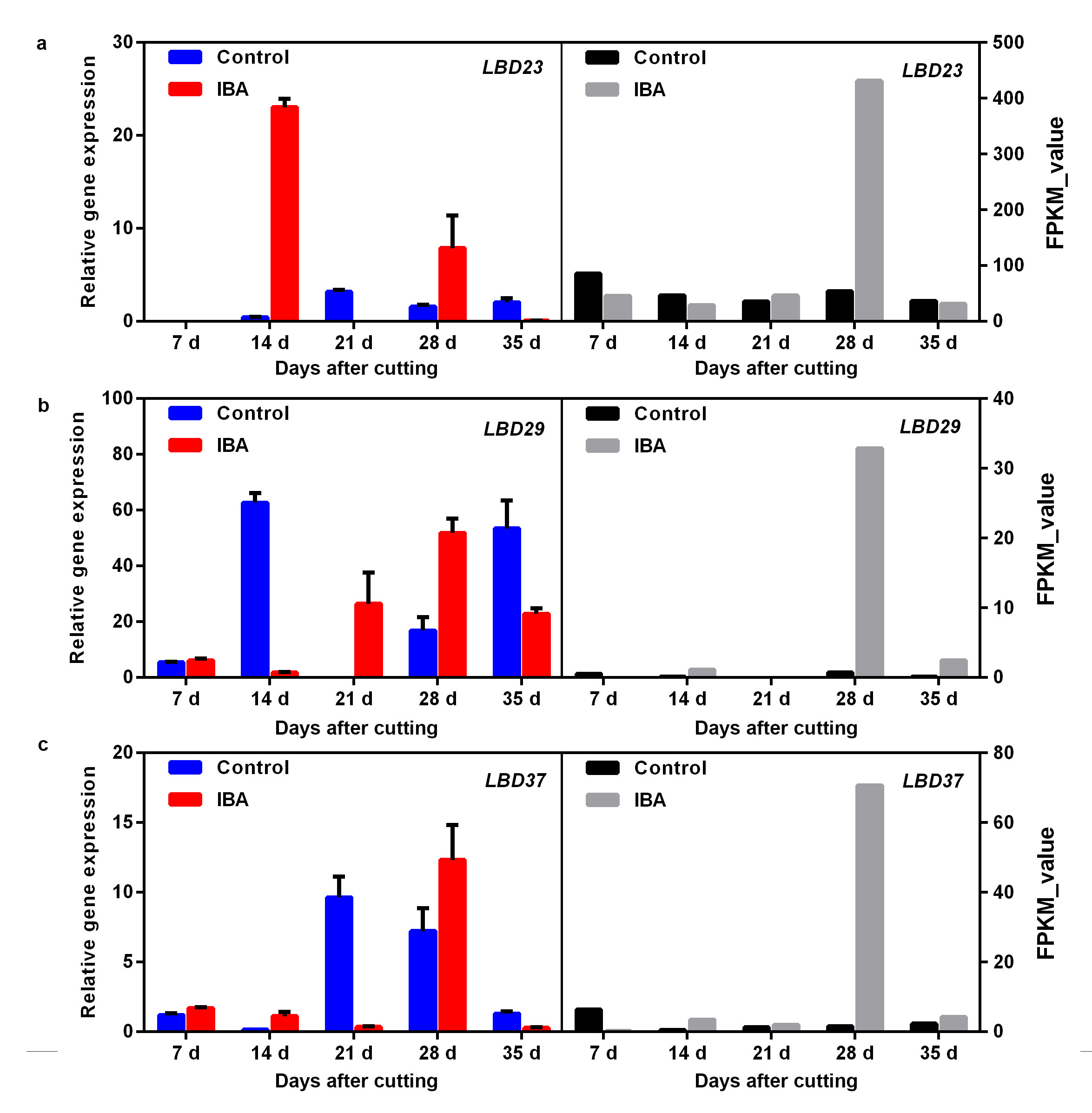

Supplement: Supplementary file 7 — Additional file 7: Figure S7. Expression of four LBDs during AR formation in blueberry green cuttings. Note: Control and IBA indicate the treatment used in this study. Axis X indicates the sampled time, e.g., 7d means the samples were collected 7 days after cutting. The bar indicates the standard error (n = 3). [file 12870_2020_2398_MOESM7_ESM.jpg]

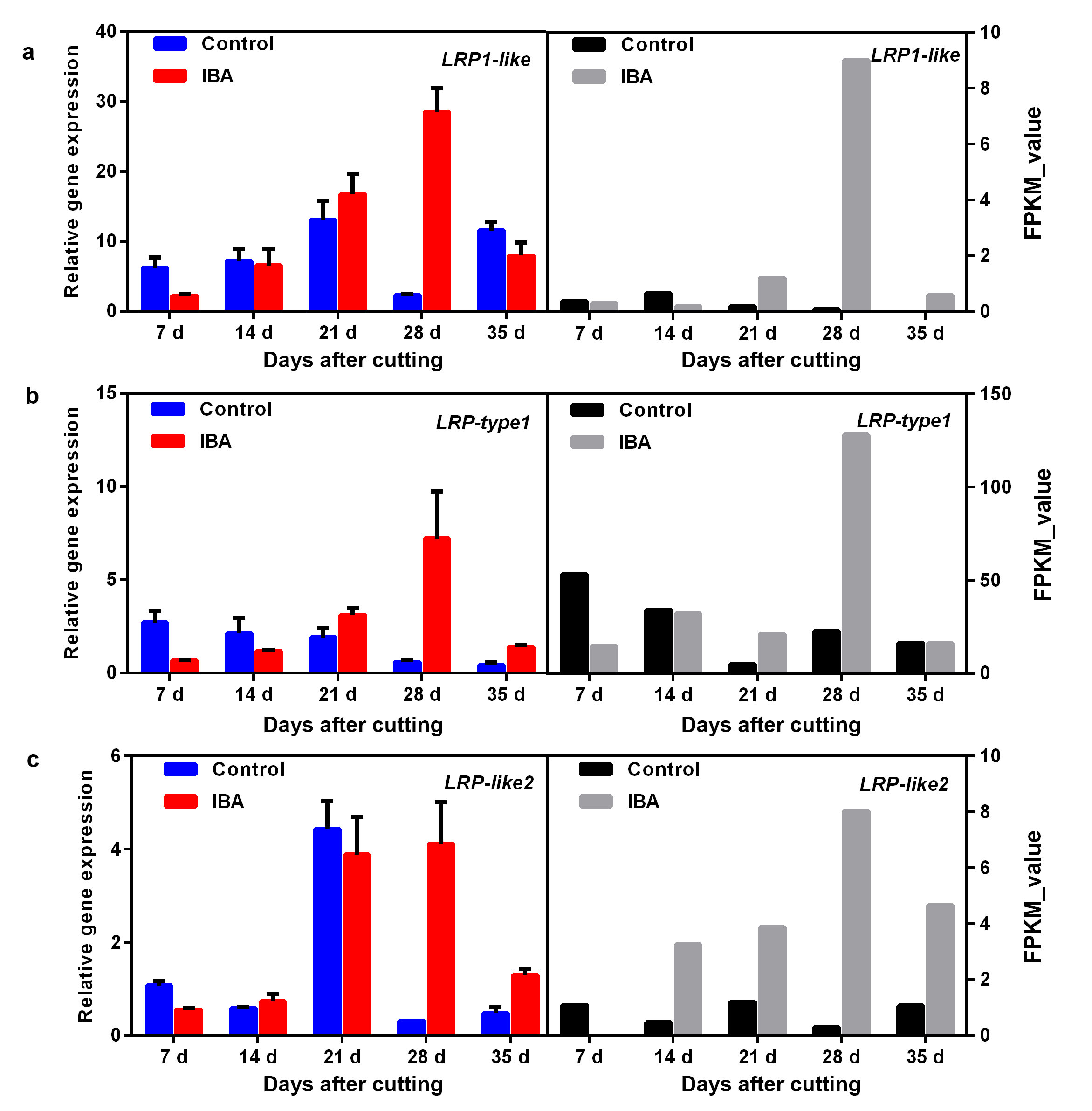

Supplement: Supplementary file 8 — Additional file 8: Figure S8. Expression of four lateral root primordium protein-related LRP genes during AR formation in the stem of blueberry green cuttings. Note: Control and IBA indicate the treatment used in this study. Axis X indicates the sampled time, e.g., 7d means the samples were collected 7 days after cutting. The bar indicates the standard error (n = 3). [file 12870_2020_2398_MOESM8_ESM.jpg]
