# Supplementary material for: Transcriptomic profiling and discovery of key genes involved in adventitious root formation from green cuttings of highbush blueberry (Vaccinium corymbosum L.)
Source: BMC Plant Biol. 2020 Apr 25;20:182. doi: 10.1186/s12870-020-02398-0 (PMC7183619; doi:10.1186/s12870-020-02398-0)
Supplement: Supplementary file 9 — Additional file 9: Table S1 Regression equation of the calibration curves for IAA, ABA, GA3 and zeatin. [file 12870_2020_2398_MOESM9_ESM.docx]

Supplementary Table 1 Regression equation of calibration curves for IAA, ABA, GA3 and Zeatin

| Hormones | Calibration curves | Correlation coefficient (*R*^2^) |
| --- | --- | --- |
| IAA | y=1477.98477x+2156.18101 | 0.99798 |
| ABA | y=29986.05852x+11579.31653 | 0.99922 |
| GA3 | y=6935.42573x+3400.73021 | 0.99966 |
| Zeatin | y=28700.80315x+671.07106 | 0.98949 |
