# Supplementary material for: Transcriptomic profiling and discovery of key genes involved in adventitious root formation from green cuttings of highbush blueberry (Vaccinium corymbosum L.)
Source: BMC Plant Biol. 2020 Apr 25;20:182. doi: 10.1186/s12870-020-02398-0 (PMC7183619; doi:10.1186/s12870-020-02398-0)
Supplement: Supplementary file 10 — Additional file 10: Table S2 Primers for the qRT-PCR analysis used in this study. [file 12870_2020_2398_MOESM10_ESM.docx]

Supplementary Table 2 Primers for qRT-PCR analysis used in this study

| Primers | Sequence (5’ to 3’) | Primers | Sequence (5’ to 3’) |
| --- | --- | --- | --- |
| *DRMH3_Fwd* | CCTTGGGAAACTGCGTAAAC | *ARF9_Fwd* | CGTGGCCAGAGTTCTGTAAT |
| *DRMH3_Rev* | CGTCGGAGGTTTCACTATCA | *ARF9_Rev* | TGGTTCCTTCGCCTTCTAATG |
| *RGF9_Fwd* | GAAAGAAAGAATGGCCGTGTTG | *ARF7_Fwd* | TGCCAAGCATCTCCATCAA |
| *RGF9_Rev* | CAGCTCCTCCATAACCAGTTTC | *ARF7_Rev* | TGAAGTTCCTGGACCACATTAG |
| *AUX22_Fwd* | GGGAGGCATTGAAGGATGAA | *PIL6a_Fwd* | CTACTACCTGCTGCCATAGTTT |
| *AUX22_Rev* | ACATCTCCCAAGGAACATCAC | *PIL6a_Rev* | ACTAGGAACTTCGGGTCTGATA |
| *LAX3_Fwd* | AAGGAATGTTGGTGGTCTCTC | *PIL6b_Fwd* | AGCTACAAGCCTCACAAGTATC |
| *LAX3_Rev* | CTCGTCAGCTTCACCGTTTA | *PIL6b_Rev* | GGCAGGTCGTGCTAAGATTAT |
| *LAX5_Fwd* | TTTGCTCTCCTCCCAACATC | *PIL6c_Fwd* | GGCAGAGGTGTGCGAAATA |
| *LAX5_Rev* | GCCTTCTCCCACACGAAATA | *PIL6c_Rev* | GCATGAGGCTGTACCTACAAA |
| *LRP-like_Fwd* | CCGGTAGTAGTGGGACAATTAG | *LBD16_Fwd* | CATGCCACCTGTTGTTGTAAAG |
| *LRP-like_Rev* | GTGCTCTTGACATGGGTTTG | *LBD16_Rev* | GCTGTGGTCACAATTGCTTATG |
| *LRP-like2_Fwd* | CCACTGGTACTTGCAGATCAA | *LBD23_Fwd* | ACACTTCCGTTCCATCATATCC |
| *LRP-like2_Rev* | TGCCAAACGCCCTCAATA | *LBD23_Rev* | CCATGGCCTTGGAAGACTTTA |
| *LRP-type1_Fwd* | CCACCTCAGTTTCGCTTTCT | *LBD29_Fwd* | GCCAGAGCCTGTCATCTTAAT |
| *LRP-type1_Rev* | TTCTGCTGCTGCTTCTTCTC | *LBD29_Rev* | TCCCTCTCCCTCTTGTGTTTA |
| *LRP1_Fwd* | AGAGAGAGAGAGAGGAGAGAGA | *LBD37_Fwd* | CGCCAATCAACCAGTAGACTAA |
| *LRP1_Rev* | CTTCGCAAACTACCACCAATTC | *LBD37_Rev* | CCCACAATCACCAAACCAAAC |
| *GAPDH_Fwd* | ACTACCATCCACTCTATCACCG |  |  |
| *GAPDH_Rev* | AACACCTTACCAACAGCCTTG |  |  |
